# Diet Modification in Saudi Women Living With Cardiovascular Disease: A Qualitative Study of Barriers and Motivators

**DOI:** 10.1097/jnr.0000000000000755

**Published:** 2026-06-29

**Authors:** Afnan TUNSI, Lujain SALLAM, Reem SINNARI, Rahaf ALGHAMDI, Rahaf ALAFGHANI, Raghad ASSIRI, Anwar TAMMAR

**Affiliations:** 1 Faculty of Nursing, King Abdulaziz University, Jeddah, Saudi Arabia

**Keywords:** cardiovascular disease, diet modification, women, qualitative research

## Abstract

**Background:**

Cardiovascular disease (CVD) is the leading cause of mortality worldwide and a major contributor to reduced quality of life. The disproportionately high risk of CVD faced by Saudi women compared with Saudi men is driven by rising rates of obesity, physical inactivity, and lifestyle-related risk factors. Despite the importance of dietary modification in secondary prevention, adherence to a heart-healthy diet remains challenging for many Saudi women due to gendered caregiving responsibilities, limited autonomy in food choices, and sociocultural expectations to prioritize family needs over their health.

**Purpose:**

This study was designed to explore the barriers and motivators related to healthy diet adherence among Saudi women living with CVD, with a particular analytical focus on how gendered household roles and sociocultural expectations shape dietary behaviors.

**Methods:**

A qualitative descriptive design was employed, involving individual semistructured interviews with 19 Saudi women. Thematic analysis was conducted in accordance with Braun and Clarke’s six-phase approach.

**Results:**

The factors identified in the analysis were grouped into four themes: individual, social, institutional, and environmental. Gender roles, particularly the responsibilities of women as primary caregivers and meal preparers, consistently shaped the ability of the participants to adhere to healthy diets. Disease severity and family support were identified as key motivators, while the identified barriers included prioritizing family preferences over personal health, lack of tailored dietary counseling, and limited access to healthy food options.

**Conclusions:**

The findings underscore the multifaceted challenges Saudi women face in adhering to heart-healthy diets, particularly those rooted in gendered caregiving roles, social obligations, institutional gaps in dietary support, and environmental barriers to food access. Effective dietary interventions must go beyond individual education and be purposefully embedded within culturally sensitive, gender-responsive strategies that address these broader structural and contextual constraints.

## Introduction

Cardiovascular disease (CVD) remains the leading cause of mortality worldwide and a major contributor to reduced quality of life, especially in developing regions (Lindstrom et al., 2022). Rising CVD-related deaths are largely attributed to population ageing, urbanization, and shifts in dietary and lifestyle patterns (Benjamin et al., 2019). In 2021, CVD was responsible for an estimated 20.5 million deaths globally, accounting for nearly one-third of all deaths (Lindstrom et al., 2022). Notably, women are significantly affected, with ∼9.6 million women dying from heart and circulatory diseases in 2021, highlighting the substantial burden of CVD among females globally (British Heart Foundation, 2023). In addition, the global age-standardized prevalence of CVD in women was estimated at 6,403 per 100,000, with a mortality rate of 204 per 100,000 (Vervoort et al., 2024). Given the substantial burden of CVD, there is a clear need for the development of scalable, evidence-based strategies, including dietary modification, to prevent the onset and progression of CVD.

In this context, dietary modification, defined as the adoption of a nutrient-rich, heart-healthy eating pattern low in saturated fats and added sugars, is foundational to CVD management strategies centered around Mediterranean, DASH, and plant-based diets, which have shown cardioprotective effects (Diab et al., 2023; Estruch et al., 2018; Lichtenstein et al., 2021). Dietary modification plays a dual role in prevention, helping in primary prevention to reduce the risk of developing CVD in healthy individuals by addressing modifiable risk factors such as hypertension, dyslipidemia, and obesity (Arnett et al., 2019). In secondary prevention, the goals of dietary modification include preventing disease progression, reducing the risk of recurrent cardiac events, and improving quality of life in patients with CVD. In combination with other behavioral interventions such as smoking cessation and physical activity, a heart-healthy diet has been shown to reduce the burden of coronary artery disease significantly (Virani et al., 2023).

In Saudi Arabia, CVD contributes to more than 45% of all deaths, with lifestyle-related factors, particularly poor dietary patterns, being a major contributor to its high prevalence (Gagnon-Arpin et al., 2018). Saudi women face unique health challenges related to cardiovascular risk due to factors such as increasing rates of obesity, sedentary lifestyle, and poor dietary habits (Alshaikh et al., 2022). Obesity rates in Saudi women range from 29.5% to 58.3% (Alhakbany et al., 2018; Alshaikh et al., 2016). Physical inactivity is alarmingly high within this group (52.1%–98.1%) and significantly exceeds the rate reported for Saudi men (Albawardi et al., 2016). Recent studies on dietary patterns among Saudi women have revealed a diverse range of eating habits influenced by sociodemographic factors and lifestyle choices. Common patterns include traditional diets high in dates, coffee, and meat (Bawazeer et al., 2021; Bukhari & Header, 2024) and western-influenced diets rich in refined grains and sweets (Aljahdali & Bawazeer, 2022). Moreover, the risk of CVD in Saudi women is compounded by sociocultural expectations and longstanding structural barriers to health care access. The ability of women to maintain healthy routines, including attending gyms and regular medical appointments, has long been hindered by legal and logistical restrictions requiring male guardianship or transportation support (Alhejely et al., 2023). Recent societal reforms, most notably the lifting of the driving ban, increased workforce participation, and a rising number of women in leadership positions, have eased some of these barriers and may have long-term implications for women’s health outcomes (Tash & Al-Bawardy, 2023).

Nevertheless, despite such recent advances, adherence to dietary recommendations remains suboptimal among women with CVD. In Saudi Arabia, national surveys have consistently shown that women with chronic diseases, including CVD, struggle to follow heart-healthy dietary patterns despite receiving general dietary advice (Alshaikh et al., 2022). A range of individual and contextual barriers, including limited nutrition knowledge or willpower (Espejo et al., 2018; Pinho et al., 2017), economic barriers that restrict access to healthy food (Chang et al., 2021), and time constraints arising from women’s dual roles in work and family caregiving (Alshaikh et al., 2022), complicate their ability to maintain a heart-healthy diet (Diab et al., 2023). In addition, sociocultural influences such as family food preferences and deeply rooted culinary traditions have been identified as challenges to dietary adherence (Barolia et al., 2019; Tunsi et al., 2023). Compounding these issues is a lack of individualized, culturally sensitive dietary guidance generally available from health care providers, with many women reporting receiving vague or inconsistent information (Seixas et al., 2020; Sterling et al., 2018).

However, several motivators of dietary change efforts in Saudi women have been identified, including encouragement from supportive social networks, physical health improvements, symptom alleviation, and the desire to avoid future health deterioration (James et al., 2022; McLean et al., 2021; Stevenson et al., 2018).

Despite the global emphasis on promoting healthier choices and reducing the risks associated with CVD, few studies have been published on the lived experiences of Saudi women attempting to adopt dietary modifications after receiving a CVD diagnosis. Given the unique cultural, social, and religious contexts of Saudi Arabia, qualitative studies are essential to understanding the nuanced barriers and motivators that influence the dietary behavior of this population. Qualitative approaches enable the deeper exploration of how gender roles, social norms, institutional interactions, and environmental conditions intersect to shape dietary decisions (Creswell & Poth, 2016). This qualitative study was designed to address this gap by exploring the barriers and motivators influencing healthy eating behaviors among women with CVD in the country, with a particular focus on analyzing the influence of gendered household roles and sociocultural expectations on dietary behavior. The findings of this study are intended to inform the development of culturally appropriate, gender-responsive strategies aimed at enhancing secondary prevention and improving cardiovascular health outcomes among Saudi women.

## Methods

### Study Design

A descriptive qualitative approach (Bradshaw et al., 2017) utilizing semistructured interviews was employed to gain an in-depth understanding of the experiences of women attempting to make dietary modifications after CVD diagnosis. This approach helped gain insight into a poorly understood research area and facilitated a subjective interpretation of textual data (Doyle et al., 2020).

### Sampling and Sample Size

The participants comprised 19 women living with CVD aged between 39 and 70. Purposive sampling using a maximum variation technique (Creswell & Poth, 2016) was employed to ensure diversity in age, socioeconomic background, and educational levels. Inclusion criteria were: (1) being a Saudi woman over the age of 18 years old, (2) having been diagnosed with CVD for at least two years (specifically atherosclerotic coronary artery diseases, including myocardial infarction, acute coronary syndrome, unstable angina, and cardiomyopathy), and (3) willingness to participate in face-to-face or phone interviews. Women diagnosed with stroke or heart failure were excluded from the study due to potential physical limitations that may significantly influence lifestyle choices.

The study was conducted in the cardiac clinics of two major hospitals between January and April 2023. Both institutions provide free inpatient and outpatient care. Data collection involved individual face-to-face interviews conducted in Arabic by five of the authors. All of the interviewers received structured training in qualitative interviewing techniques from the first author, who is an experienced qualitative researcher with graduate-level training in qualitative methods. This training included simulated interviews with role play as well as guided feedback, ethical considerations, and standardized probing techniques to ensure consistency across data collection. Before interviewing independently, each coauthor completed at least one observed mock interview and one pilot interview, with feedback provided by the first author. An interview guide with open-ended questions was developed based on the existing literature and the first author’s experience. The guide was reviewed by all of the interviewers, and a consensus was established using pilot testing and debriefing sessions. The team met regularly during the early stages of data collection to discuss interview flow, adjust prompts when necessary, and ensure alignment in approach. The interview guide is outlined in Table 1.

**Table 1:** Semistructured Interview Guide

No.	Question
Initial interview questions	
1.	How long have you been diagnosed with cardiovascular disease?
2.	What do you know about your disease and its relationship to diet?
3.	Do you believe in the importance of a healthy diet and its impact on heart disease?
4.	Were you given a healthy diet plan after your diagnosis?
5.	Tell me about the care and information you received from health care providers (doctor, nurse, dietitian)?
Experience questions	
1.	Tell me about the changes that occurred in your life after diagnosis?
2.	Are you having difficulties choosing the appropriate food?
3.	What difficulties did you face in adhering to a healthy diet? In all aspects, whether (financial, psychological, or environmental)?
4.	What factors motivate you to stick to a healthy diet?
5.	After you were diagnosed with cardiovascular disease, did family members change their eating patterns to accommodate your health condition?
6.	If the answer is yes, what kind of change did the family members make?
7.	What are the motivations you received from the health care center you are attending, or from your family?
8.	What is the impact of your family and the community around you on your food?
9.	Were you involved in the dietitian’s plan? Or Did you have a decision in your diet plan?
10.	If the information that the doctor gave you about the diet was not enough for you, what are the sources that you resort to to gain information?
11.	What is the positive aspect that you see if you adhere to a healthy diet?
12.	In case you are following up with a dietitian, could you please tell me what are the food plans/diets that have been prescribed for you?
13.	What is your current diet?
14.	Are there any gender-specific barriers and motivators for diet change?
Ending questions	
1.	In your opinion, what are the changes and improvements that can help the patient adhere to a healthy diet?
2.	What advice would you give to someone with cardiovascular disease?

Each interview lasted between 30 and 45 minutes and was digitally recorded to provide an accurate account of participant perspectives. Field notes were collected to augment the data and enrich contextual understanding. Clinic nurses acted as gatekeepers in the process of recruiting eligible participants. Data saturation was achieved after 17 interviews, as no new codes or themes were identified, with subsequent interviews confirming previously established categories and not contributing novel insights. To ensure rigor, two additional interviews were conducted beyond the point of saturation to verify thematic consistency (Guest et al., 2006). Saturation was determined inductively during ongoing analysis, consistent with the approach of Braun and Clarke (2006) to thematic analysis.

### Data Analysis

Analysis followed the thematic analysis six-step approach by Braun and Clarke (2006) and was carried out simultaneously alongside the data collection process to allow iterative reflection and refinement. All of the interviews were transcribed verbatim in Arabic and translated into English by bilingual members of the research team. A backwards translation was performed by an independent professional translator to ensure accuracy of meaning. Familiarization with the data was achieved by the primary coders repeatedly reading transcripts and then performing line-by-line coding manually. Codes were generated inductively and grouped into subthemes, which were then organized into four overarching themes, namely individual, social, institutional, and environmental. The level of influence assessment was guided by the socioecological model by McLeroy et al. (1988), which emphasizes the interaction of personal and environmental factors in shaping health behaviors. This model is commonly used in public health research to contextualize behavioral determinants. Thematic development and refinement were achieved through the conduct of multiple analytic meetings with the full research team to ensure consistency and representativeness across the data set. To enhance rigor, coding consistency was verified using peer checking. After initial coding, 20% of the transcripts were independently coded by three researchers not affiliated with this project, with discrepancies resolved through consensus. A coding audit trail was maintained throughout to document coding decisions and theme revisions, and all team members involved in analysis work had prior training in qualitative research, further supporting the trustworthiness of the findings.

### Trustworthiness

The four criteria of credibility, transferability, dependability, and confirmability were used to enhance methodological rigor (Yadav, 2022). Data credibility was maintained through prolonged research team engagement with the data set and by conducting frequent analytic meetings to discuss emerging themes and interpretations. Although member checking and triangulation were not used due to logistical constraints, analytic rigor was maintained using collaborative coding and consensus-building during team discussions. Transferability was addressed in this study by providing a detailed description of the context and participant characteristics, including age (range: 39–70 years), socioeconomic and educational backgrounds, and types of CVD diagnosis. Direct quotations from participants were used throughout to convey the richness and depth of their lived experiences. Dependability was enhanced by maintaining an audit trail that documented coding decisions, theme revisions, and methodological reflections. The team conducted peer debriefings to review each step of the analytic process critically and ensure consistency over time. Lastly, confirmability was achieved through reflexivity. Field notes were used by researchers to document personal assumptions, preconceptions, and emotional responses during data collection and analysis. This reflective process helped minimize bias and enhance the neutrality of interpretations. Collectively, these strategies enhanced the rigor, reliability, and transferability of the qualitative findings of this study.

### Ethical Considerations

This study complies with the Declaration of Helsinki (World Medical Association, 2013). Ethical approval was obtained from the Nursing Research Ethical Committee of the Faculty of Nursing at King Abdulaziz University in Jeddah (Ref No. 3B.07) as well as from the ethics committees of both recruitment hospitals (IRB No.: A01535). Informed consent was obtained from each participant, and all received clear explanations of the study purpose, procedures, and potential risks (Polit & Beck, 2021). The privacy and confidentiality of the participants were maintained through the use of a secure data storage laptop with encrypted passwords and participant anonymization. The risk of disclosing personal and sensitive information was discussed. Participants were assured of voluntary participation, the right to withdraw at any time, and that withdrawal would have no influence on their medical care.

## Results

**Figure 1:**
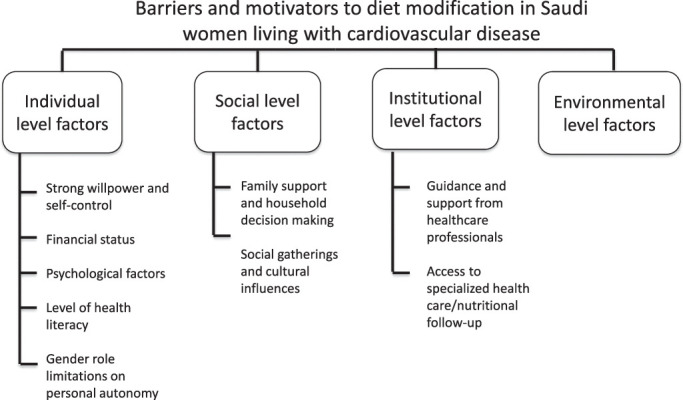
Summary of Themes and Subthemes

In-depth semistructured individual interviews were conducted with 19 participants aged between 39 and 70 years. Demographic details collected included age, type of cardiovascular disease, occupation, educational level, and income (Table 2). A wide range of barriers and motivators were found to influence the ability to adopt and maintain a heart-healthy diet. A central analytical focus on gendered household roles and sociocultural expectations informed the interpretation of participant experiences across all levels of influence. The findings were categorized into the four major themes of individual-level factors, social-level factors, institutional-level factors, and environmental-level factors. Each theme was further divided into subthemes, as illustrated in Figure 1, and supported by quotations from participants, which are presented in italics.

**Table 2:** Participant Demographics

Code	Age	Type of CVD	Occupation	Educational Level	Level of Income	Residential Area	Marital Status
P1	67	UA	Housewife	High school	Medium	Urban	Married
P2	58	MI	Housewife	Intermediate school	Medium	Urban	Married
P3	50	ACS	Employed	Bachelor	High	Urban	Married
P4	77	MI	Housewife	Illiterate	Medium	Urban	Widow
P5	76	MI	Housewife	Illiterate	Low	Urban	Married
P6	70	MI	Housewife	Illiterate	Low	Urban	Widow
P7	65	ACS	Housewife	Illiterate	Medium	Urban	Married
P8	42	Cardiomyopathy	Employed	Bachelor	Medium	Urban	Married
P9	54	ACS	Housewife	Middle school	Medium	Urban	Married
P10	50	ACS	Housewife	Illiterate	Low	Urban	Married
P11	62	ACS	Housewife	Elementary school	High	Urban	Married
P12	63	ACS	Housewife	High school	Medium	Urban	Married
P13	41	Cardiomyopathy	Employee	High school	Medium	Urban	Married
P14	39	MVS	Housewife	High school	Medium	Rural	Married
P15	67	MI	Housewife	Illiterate	Medium	Rural	Married
P16	58	Cardiomyopathy, MI	Housewife	Diploma	High	Urban	Widow
P17	53	ACS	Housewife	Illiterate	Low	Urban	Widow
P18	62	MI	Housewife	Elementary school	Medium	Rural	Widow
P19	71	MI	Housewife	Illiterate	Medium	Urban	Married

*Note*. CVD = cardiovascular disease; UA = unstable angina; MI = myocardial infarction; ACS = acute coronary syndrome; MVS = multivessel disease.

### Theme 1: Individual Level Factors

Individual-level factors included five subthemes: (1) strong willpower and self-control, (2) financial status, (3) psychological factors, (4) level of health literacy, and (5) gender role limitations on personal autonomy. Throughout this theme, the narratives reflected how participants’ roles as caregivers, housewives, or employed women shaped their food choices and constrained their autonomy over diet-related decisions.

#### Subtheme 1.1: Strong willpower and self-control

Many of the participants cited strong willpower and self-control as motivators in their commitment to a healthy diet, especially in challenging social contexts such as weddings and family gatherings. One participant noted: *“At gatherings and weddings, I don’t eat. When I come home, my daughter makes my dinner, or I bring my food.”* (P4) *“I accept the idea that I cannot change their food choices. When I go there, I eat, but in small quantities, and sometimes I wait until I get home and eat my healthy food.”* (P13)

#### Subtheme 1.2: Financial status

Many participants identified financial constraints as a barrier to maintaining a heart-healthy diet. Regardless of employment status, women described struggling to afford nutritious food. For some, these financial difficulties were compounded by their caregiving responsibilities or dependence on other household members for food-related decisions. “*My financial situation is one of the major factors preventing me from changing or maintaining my diet. I do not have enough money to buy healthy food just for myself.”* (P10) “*Honestly, you know how expensive it is to buy healthy food, so we eat from what’s available*” (P17) “*Healthy food needs a certain budget.*” (P18)

#### Subtheme 1.3: Psychological factors

Participants described how both physical and psychological distress influence their dietary behaviors. Physical symptoms such as fatigue, discomfort, and worsening health conditions were commonly cited as motivators for adopting a heart-healthy diet. For example, one participant stated, “*I started committing during the last two months when my health deteriorated.”* Another explained, *“When I eat fatty food, I get tired, and I feel like there’s a burning sensation here, like a fire. And I get lethargic.”* (P10) These symptoms reinforced the importance of following dietary recommendations.

In contrast, many participants expressed that psychological challenges such as anxiety, sadness, and fear often hindered their adherence to a healthy diet. Fear was shown to serve a dual role in terms of sometimes encouraging healthy eating and at other times causing emotional stress and confusion around food choices. As one participant shared, *“I eat healthy because I am anxious about my health.”* (P11) However, others described how fear and uncertainty prevented them from eating altogether: *“Sometimes, I crave food, but I don’t eat it. I am so scared now to eat anything that may affect my heart and make me worse.”* (P4)

These comments illustrate the complex role of psychological health in lifestyle management and how psychological distress may both motivate and hinder dietary adherence.

#### Subtheme 1.4: Level of health literacy

Level of health literacy emerged as a factor influencing the ability to adopt and sustain dietary modification. Several participants stated their limited literacy hindered their understanding and adherence to heart-healthy dietary recommendations. Some described being unable to read the standardized printed dietary instructions provided by health care professionals, which were not tailored to their literacy level or socioeconomic status. One participant explained: “*The nutritionist gave me a schedule, but my problem is that I cannot read. I show it to my daughter, but she always forgets; she also works and is mostly busy.”* (P10)

In contrast, those participants with higher levels of health literacy expressed proactive behaviors in seeking nutrition-related knowledge beyond what was provided during consultations. They actively searched for information on foods that may negatively impact their condition and demonstrated critical thinking about their dietary decisions. As one participant shared: *“I was always looking for the bad food choices that the nutritionist did not mention to me. So I searched for them to see whether they are harmful to me or not.”* (P13)

These contrasting experiences highlight how varying levels of health literacy influence dietary self-management among Saudi women. While some of the participants relied on others due to literacy limitations, others demonstrated autonomy and motivation through self-education.

#### Subtheme 1.5: Gender role limitations on personal autonomy

Considering the focus of this study on women, gender-specific limitations emerged as a subtheme that primarily reflected constraints on personal time, mobility, and dietary autonomy. Participants described juggling employment, childcare, and household tasks, which left them insufficient time to focus on their own dietary needs. *“I prepare food for the entire family, and most of the time I don’t have enough time to cook for myself. This is the most difficult thing I have faced.”* (P3) Another participant noted: *“I am an employee, when would I be able to prepare healthy food for myself?”* (P6)

In Saudi Arabia, women are traditionally expected to take on the primary role of preparing meals for their families. This expectation generates time pressure and role overload, making it difficult to maintain a healthy diet. *“I’m always preparing food for my kids, and I don’t have time to think about what I should eat.”* (P7)

Notably, the intensity and nature of these limitations were shown to vary across socioeconomic status. Those participants from lower-income households expressed greater challenges in affording healthy food, which compounded time pressures and limited options for personal meal preparation.

In contrast, women with higher educational or income levels, for example, employed professionals and those with supportive partners, reported having slightly more flexibility in shaping household diets. Some of them described delegating meal preparation, using delivery services for healthier options, or consciously preparing separate meals for themselves when necessary. However, even in these cases, caregiving and hosting expectations still influenced their behaviors. “*Sometimes I cook for my family and prepare something different for myself because I know what I can eat. But it takes effort.”* (P8)

*“At least I can order healthy food when I don’t have time to cook, but it’s not always affordable.”* (P13)

In addition, some of the participants also reported challenges related to autonomy in accessing healthy food choices due to traditional household structures. They explained they could not easily go out to purchase healthy food, either due to transportation limitations or social norms. *“Men can go out whenever they want and eat everything. They are free, unlike women who will have a hard time going out or buying the food they want.”* (P15)

Even the unemployed participants, despite having more time, reported similar struggles, largely due to the continuous demands of childcare and family-centered food habits. One participant shared, *“Since I am unemployed and there is nothing I can do other than taking care of my kids, I eat everything, I eat with my young kids; and I don’t think whether it is healthy or not. All I think about is that I need to feed my kids and eat what they like.”* (P11)

These findings highlight how gender role expectations intersect with socioeconomic status to shape women’s dietary behaviors. Addressing these barriers requires tailored, culturally sensitive interventions that take into consideration financial limitations, caregiving roles, and societal norms. While framed as an individual-level theme, gendered roles were also shown to interact with social, institutional, and environmental barriers, reinforcing their complexity and pervasiveness in the everyday lives of the participants. The interpersonal influences from spouses and children are presented in Subtheme 2.1.

### Theme 2: Social Level Factors

This theme explores how family dynamics and cultural contexts shape dietary behaviors and includes two subthemes: (1) family support and household decision making, and (2) social gatherings and cultural influences.

#### Subtheme 2.1: Family support and household decision making

Family was shown to play a dual role in terms of sometimes enabling dietary adherence and other times presenting obstacles and acting as a barrier. The focus of this subtheme is on interpersonal processes, which cover the behaviors of spouses and children and household decision dynamics. Thus, this subtheme is distinct from the individual role constraints detailed in Subtheme 1.5. The participants emphasized the importance of household support, noting that positive reinforcement and shared participation in dietary changes increased their motivation. This was especially true when other family members were also affected by heart disease. As one woman shared: *“It depends on the family. If they accept the change, they will help you to adopt it for the rest of your life. But if they do not accept this, then the matter will be difficult for you.”* (P3) Another explained: “*They [family] motivate me more to eat healthy, and they all want to eat healthy options.”* (P12) In addition, for some participants, adhering to a healthy diet was positively influenced by previously affected family members with heart disease: “*My mother has a heart condition. When I see her tired with the progression of her disease, I stick to healthy eating and exercise so that I do not reach what she has reached.”* (P3)

Conversely, household decision processes were sometimes shown to undermine the efforts of participants to sustain healthy habits when spouses or children resisted change or controlled purchasing. Some described husbands who would not modify their own eating behaviors or who purchased unhealthy items: “*My financial situation affects what I can cook. If my husband brings unhealthy food, that’s what I cook. I can’t force him to buy things he doesn’t want.”* (P10) Another participant noted: “*He [husband] does not eat any of the food that I eat. He is a picky eater, he does not like fish, and eats plenty of junk food.”* (P16)

Financial and menu gatekeeping also limited the feasibility of separate meals: “*I eat what’s available for everyone. We can’t afford different meals for me.”* (P17) Children’s preferences also pulled diets toward restaurant food: *“My kids love to eat food from restaurants. Sometimes they eat what I cook, but most of the time they don’t.”* (P18) These barriers are interpreted as a lack of interpersonal support or household gatekeeping rather than as role constraints.

#### Subtheme 2.2: Social gatherings and cultural influences

The participants reported avoiding social gatherings due to dietary restrictions and fear of health complications. This was often found to promote social withdrawal and a sense of isolation. One participant noted: “*Before I got sick, I used to go everywhere and do whatever I wanted without needing anyone. Now I am afraid of its recurrence or me collapsing, so I stopped going alone.”* (P15) Cultural norms surrounding hospitality were shown to place additional pressure on the participants to conform to expected behaviors, including consuming food that may not align with their dietary needs. In Saudi society, declining food offered in social settings is often perceived as disrespectful—an expectation that disproportionately affects women, who are expected to uphold traditions of politeness and gratitude: “*But you have to eat because it is a shame that the food is prepared for you and you do not eat.”* (P12)

*“No, I eat what is available, and I do not ruin my diet. I do not burden people; one should respect people. We believe that if you respect people, they will respect you back.”* (P10)

These examples illustrate how sociocultural expectations rooted in gendered socialization intersect with interpersonal dynamics and constrain the autonomy of Saudi women over their dietary choices, forcing them to navigate between cultural obligations and health needs. Gendered norms around hospitality, caregiving, and self-sacrifice emerged as key forces shaping food behaviors in social contexts.

### Theme 3: Institutional Level Factors

This theme, which describes how the participants engaged with health care professionals and the support they received, includes two subthemes: (1) guidance and support from health care professionals, and (2) access to specialized health care/nutritional follow-up.

#### Subtheme 3.1: Guidance and support from health care professionals

Many of the participants reported that the guidance they received from health care professionals lacked specificity and personalization. A common concern was that these professionals assume women are inherently knowledgeable about food preparation due to traditional gender roles. This assumption often leads to the provision of brief, generalized advice without consideration of the literacy level, caregiving burden, or financial limitations of the advice recipient.

*“I did not get a specific diet plan. Instead, the physician just reminded me about cutting down on fats and carbohydrates and maintaining my weight.”* (P1)

*“They did not give me a specific food plan, but advised me to stay away from salt and eat boiled food.”* (P7)

These interactions suggest that the central role of women in household nutrition is taken for granted, therefore diminishing the perceived need for structured dietary counseling. Despite feeling generally satisfied with physician communications, the participants expressed frustration that dietary advice is often overshadowed by a stronger emphasis on medication adherence:

*“During follow-ups, they do not talk about food every visit, and the cardiologist only stresses compliance with medications.”* (P5) Medical-based approaches that prioritize pharmacological interventions may unintentionally perpetuate gender-based disparities in dietary counseling by failing to account for the practical challenges that women face in implementing long-term dietary change.

#### Subtheme 3.2: Access to specialized health care/nutritional follow-up

The participants consistently highlighted the limited involvement of dietitians during both hospitalization and follow-up care. When dietitians were consulted, their advice was often vague and duplicative of what physicians had already stated:

*“She [the dietician] talked to me only once to check if I followed the instructions, that’s all.”* (P13) *“The dietitian told me the same things the doctor told me - don’t eat oily and salty food. She also advised me to move and walk.”* (P16)

This lack of individualized nutritional planning left many participants feeling unsupported in managing their dietary changes. Over half of the participants reported not being referred to a dietitian or receiving structured follow-up. The participants attributed this lack of support to systemic challenges within the health care system, including a shortage of dietitians, insufficient institutional prioritization of nutritional care, and a health care culture that emphasizes pharmacological treatment over long-term dietary counseling. Even when nutritional services were available, they were limited in scope and frequency, and often insufficient to meet patients’ ongoing needs. *“There aren’t enough nutritionists to help everyone. The doctor just said, ‘take your medicine’ and didn’t talk much about food.”* (P8)

Even when services were available, the participants encountered practical barriers such as long waiting times and infrequent appointment availability. These structural delays were particularly detrimental in managing chronic conditions such as CVD: *“When I got out of the hospital, I used to follow up every one to two months.* *Then the doctor said, 'Your next appointment is in seven months because you are fine now. But I’m a cardiac patient. How can I wait that long?'”* (P19)

These findings emphasize that limited access to dietitians was not only a matter of individual neglect or patient motivation, but a reflection of broader systemic challenges, including workforce shortages and service prioritization. Taken together, these institutional factors significantly limited the ability of the participants to receive consistent, personalized dietary support, complicating their efforts to maintain the long-term changes essential for the secondary prevention of cardiovascular disease.

### Theme 4: Environmental Level Factors

This theme highlights how limitations in terms of the accessibility and availability of food resources posed a major challenge to participants’ adherence to a heart-healthy diet. Some noted that having access to nearby food outlets supported their efforts to eat healthily. However, this was not the case for many others, particularly for those living in rural areas. These participants reported a significant lack of food outlets, including supermarkets, diet centers, and hospitals. In many cases, even the nearest supermarket offered limited options:

*“We only have one nearby supermarket that does not have a lot of options, so that is why it is hard to follow a healthy diet.”* (P14)

This geographical constraint made it difficult for women to maintain a consistent, heart-healthy eating pattern and often required them to travel long distances to access a broader range of food items.

Importantly, these environmental limitations were further compounded by gendered restrictions on mobility and autonomy. Several participants explained they could not independently leave the house to shop for healthier food due to family expectations or lack of transportation: *“Men can go anytime to get what they want. We [women] have to ask, wait, or just make do with what’s available.”* (P15)

These narratives underscore how environmental barriers are not solely geographic or economic but also deeply influenced by sociocultural gender norms. Restrictions on women’s freedom of movement limited the ability of participants to make autonomous dietary choices and constrained their access to environments supportive of healthy eating.

## Discussion

In this qualitative study, a sociological lens was taken to examine the barriers and motivators related to dietary modification among Saudi women with CVD. The findings suggest that eating behaviors are embedded in sociocultural and structural contexts encapsulated in the four intersecting themes of individual, social, institutional, and environmental factors. To avoid thematic overlap within the socioecological framing, individual role constraints (time, mobility, personal dietary autonomy) were analyzed separately from interpersonal household influences (family support and household decision-making) to clarify distinct targets for interventions.

At the individual level, a strong motivator for dietary change was identified as the desire to achieve a longer and healthier life and was shown to be driven by strong willpower and self-control. Also, psychological distress was found to play a dual role in shaping dietary behaviors, serving as both a motivator and a barrier to heart-healthy eating. While some participants were driven to adhere to dietary recommendations out of fear of disease progression or physical symptoms, others experienced heightened anxiety levels that led to food avoidance or uncertainty around what to eat. This ambivalence reflects the emotional burdens often associated with chronic illness, particularly among women who bear both caregiving responsibilities and the internalized expectations of health maintenance. In Saudi society, women are traditionally seen as caregivers, and often prioritize the health of their family over their own well-being. This internalized duty can motivate health-seeking behaviors when women perceive their ability to care for others is at risk. While these findings resonate with the findings of Castillo-Mayén et al. (2020), who found that self-efficacy and emotional regulation influence dietary adherence, they also diverge in terms of emphasizing the culturally specific moral obligation Saudi women feel toward preserving their role in the household. Unlike in western contexts, where health autonomy is often individualistic, it is deeply relational in Saudi Arabia.

In contrast to some international studies that emphasize cost and financial issues as a primary barrier to healthy eating (Barolia et al., 2019; Pinho et al., 2017), many of the participants in this study did not view financial strain as the main obstacle. Instead, they emphasized their ability to modify traditional meals using affordable, healthier methods rather than altering food choices. This reflects the high cultural value placed on home-cooked food in Saudi Arabia. However, those from lower socioeconomic backgrounds did report struggling to prioritize dietary quality over household obligations, particularly when managing large families. This nuanced finding illustrates that financial concerns cannot be universally categorized and must be considered within the local context of traditional food customs and household responsibilities.

Health literacy factors emerged as dual influencers. Some of the participants, particularly those with higher literacy levels or support from educated family members, actively sought disease-specific nutritional information. However, others struggled to interpret standardized dietary plans due to illiteracy. These disparities align with those reported by Almubark et al. (2019), who found that nearly half of the population in Saudi Arabia has low health literacy. Thus, future dietary interventions in Saudi Arabia should prioritize inclusive communication strategies that utilize, for example, illustrated materials, audio-visual aids, and family-centered counseling, especially in cardiology and primary care settings. In addition, public health messaging should not only be linguistically accessible but also culturally and educationally adapted. Unlike settings with universally high health literacy, tailored interventions here must address a wider range of informational needs.

At the social level, family support was shown to be a powerful enabler of healthy eating, and its absence created significant challenges. This is particularly salient in Saudi Arabia’s collectivist culture, where food is a central feature of family life and communal identity. In line with previous studies (Sterling et al., 2018; Stevenson et al., 2018), positive familial influence was shown to motivate adherence to dietary restrictions, especially when shared by family members. Yet, many of the participants reported feeling isolated when their efforts were not mirrored by their spouses or children. In addition, autonomy in food choice was often limited by gender dynamics within the home, especially when male household members demanded traditional or unhealthy meals. This diverges from Western findings, which generally emphasize individual autonomy in dietary decisions.

Social gatherings, a hallmark of Saudi hospitality, were found to further complicate dietary adherence. Cultural norms regarding generosity and respect often pressure women to consume unhealthy food to avoid offending hosts. This tension between cultural belonging and medical advice promotes emotional and behavioral conflict. Similar observations were made by Tunsi et al. (2023), who noted that traditional foods carry symbolic meaning in Saudi society. Unlike in some Western contexts, where special dietary needs are increasingly accommodated, in Saudi culture, deviation from communal food traditions can be seen as disrespectful. These dynamics cannot be adequately addressed through conventional nutritional education alone. Culturally competent strategies should include messaging that redefines healthy eating as an act of social responsibility and self-respect, rather than a rejection of tradition. Although religious beliefs are an important aspect of Saudi culture and may influence dietary behaviors, this factor did not emerge explicitly in participant narratives. This absence may suggest religious dietary norms are so embedded in everyday life that they are perceived as routine rather than distinct influences on eating behavior.

At the institutional level, many of the participants received generic dietary advice informed by the assumption that women, due to their caregiving roles, already possess adequate nutritional knowledge. This assumption overlooks the significant variability in literacy, education, and caregiving burdens among women. Consistent with Seixas et al. (2020), nutritional care was deprioritized relative to pharmacological treatment, with little structured follow-up or routine access to dietitians available. Compared with high-income countries, where multidisciplinary teams are more common, the fragmented model of care prevalent in Saudi Arabia limits opportunities for tailored dietary counseling. Despite these challenges, some of the participants demonstrated strong agency by seeking dietary information independently. This proactive behavior underscores the need for more patient-centered education that goes beyond generic advice and actively engages women in their care.

Environmental factors, particularly limited food access in rural areas, further constrained the ability of the participants to maintain heart-healthy diets, aligning with evidence that access and affordability shape diet quality (Lichtenstein et al. 2021; Pinho et al. 2017). In the Saudi context, these barriers are compounded by gendered limitations in mobility. Despite recent reforms, many women still rely on male family members for transportation, reducing autonomy over food choices. This unique intersection of infrastructure and social norms is rarely addressed in international studies but has major implications for public health planning in Saudi Arabia.

Across all levels, gender roles and expectations consistently emerged as an overarching influence on dietary behavior, shaping how the participants perceived their roles, interacted with health care providers, negotiated food choices within families, and navigated public spaces. Although gendered roles and family dynamics are intertwined in practice, treating them as separate levels (intrapersonal vs. interpersonal) was shown to improve analytic clarity. These findings support calls for gender-responsive dietary interventions that take into account the lived realities of Saudi women, addressing not only their biological risk factors but also their social constraints, cultural expectations, and daily responsibilities.

### Strengths and Limitations

This study is the first conducted to investigate the perspective of Saudi women with CVD regarding adherence to a heart-healthy diet. Recruiting participants from multiple settings provided a relatively large and diverse perspective, representing a strength of this research. In addition, the use of a descriptive qualitative design facilitated the provision of detailed accounts of women’s experiences and helped in providing a thorough, in-depth understanding of their perceptions and needs to maintain a healthy diet, which accomplishes the aim of the study. Nonetheless, all research has limitations. First, this study was limited to patients with atherosclerotic CVD and cardiomyopathy, which narrows its focus. In addition, the translation of interview materials and data from Arabic to English is considered a potential limitation due to the risk of loss of meaning. However, the backward translation process used enhanced the quality of translation and the trustworthiness of the findings (Yunus et al., 2022). Finally, the participants were limited to a narrow geographical area and thus may not be representative of other areas in Saudi Arabia.

### Implications for Practice and Policy

This study highlights practical and policy-level actions that may be taken to support dietary adherence among Saudi women with CVD.

At both the individual and family levels, interventions should acknowledge women’s caregiving roles and cultural expectations. Involving family members, especially male decision-makers, in dietary education can promote shared responsibility and ease pressure on women. Also, tailoring heart-healthy modifications of traditional meals may increase acceptance and adherence.

At the clinical level, health care providers should offer individualized, culturally appropriate dietary advice. Generic recommendations fail to account for diverse literacy levels, caregiving burdens, and emotional stress. Visual tools, simplified guides, and telehealth options may be used to improve communication and reach women in rural or mobility-restricted settings. Dietitians should play a more central role in cardiac care with routine referrals and structured follow-up.

At the environmental and policy levels, improving access to healthy food is essential. Policymakers should invest in local markets, mobile nutrition services, and infrastructure to allow women greater autonomy in food purchasing. Community-based programs can enhance nutrition literacy and reduce urban–rural disparities.

Collectively, these strategies must be gender-responsive and culturally grounded to promote sustainable dietary change and improve cardiovascular outcomes.

### Conclusions

In this qualitative descriptive study, the barriers and motivators influencing dietary changes among Saudi women diagnosed with cardiovascular disease were explored and elucidated. The findings reveal how individual, social, institutional, and environmental factors intersect with gender roles and cultural expectations to shape the dietary behaviors of women in Saudi Arabia. Emotional responses, caregiving duties, limited access to tailored support, and sociocultural pressures were found to both enable and hinder heart-healthy eating. These insights underscore the importance of developing culturally sensitive and gender-responsive dietary interventions that acknowledge the lived realities of Saudi women. The findings of this study provide a foundation for designing and developing context-specific programs and policies that can support sustainable dietary change and, ultimately, improve long-term cardiovascular health outcomes.
